# Diagnostic Challenges of Medullary Carcinoma of the Small Intestine During the COVID-19 Pandemic

**DOI:** 10.3390/jcm14020298

**Published:** 2025-01-07

**Authors:** Danuta Szkudlarek, Katarzyna Skórkowska-Telichowska, Benita Wiatrak

**Affiliations:** 1 Pathology Department, Provincial Hospital Center of the Jelenia Góra Valley, 58-506 Jelenia Góra, Poland; 2 Department of Non-Surgical Clinical Sciences, Faculty of Medicine, Wrocław University of Science and Technology, Wybrzeże Wyspiańskiego 27, 50-370 Wrocław, Poland; 3 Department of Endocrinology, Jerzy Gromkowski Regional Specialist Hospital, Koszarowa 5 Str., 51-149 Wrocław, Poland; 4 Department of Pharmacology, Faculty of Medicine, Wrocław Medical University, 50-345 Wrocław, Poland

**Keywords:** medullary carcinoma, small intestine neoplasm, COVID-19 diagnostic challenges, gastrointestinal cancer, immunohistochemistry, calretinin, rare cancer diagnosis

## Abstract

**Background**: Medullary carcinoma of the small intestine is an exceptionally rare subtype of gastrointestinal cancer, characterized by its solid growth pattern and lack of glandular structures, which complicate timely diagnosis. During the COVID-19 pandemic, diagnostic delays for rare cancers became increasingly common due to the prioritization of COVID-related cases and patient reluctance to seek medical attention. **Methods and Result**: We present the case of a 70-year-old male initially misdiagnosed with COVID-19, whose persistent symptoms led to the eventual discovery of medullary carcinoma. Imaging studies revealed focal lesions in the liver, spleen, and thickened small intestinal walls, prompting surgical resection of a 16 cm intestinal segment. Histopathological examination confirmed medullary carcinoma with lymph node and liver metastases, supported by immunohistochemistry, which showed positive markers (calretinin, pancytokeratin, cytokeratin 7) and excluded other malignancies. **Conclusions**: The diagnostic delay, exacerbated by the pandemic, highlights the challenges of distinguishing rare cancers from more common conditions during global health crises. This case underscores the importance of advanced diagnostic techniques, such as immunohistochemistry, for accurate identification. Maintaining robust cancer diagnostic pathways during emergencies is crucial to avoid delays in treatment. Future research should focus on improving screening methods for rare cancers and developing resilient healthcare systems to mitigate similar challenges in future crises.

## 1. Introduction

Rare cancers represent a unique and exceptionally challenging domain in medicine, both in diagnostics and treatment. These malignancies, defined as affecting fewer than six individuals per 100,000 annually, pose distinctive hurdles due to limited clinical knowledge, nonspecific symptoms, and often scarce diagnostic and therapeutic guidelines. Within this category, small intestine cancers account for less than 5% of all gastrointestinal cancers, making them an uncommon but diagnostically complex group of malignancies [1,2]. Medullary carcinoma of the small intestine (MC-SI) is an exceedingly rare subtype, characterized by its solid growth pattern and histopathological resemblance to other poorly differentiated tumors. These features complicate its differentiation from more common gastrointestinal cancers, such as neuroendocrine tumors or poorly differentiated adenocarcinomas, frequently resulting in diagnostic delays [3].

Rare cancers are not only medically challenging but also emotionally and socially impactful for patients and their families. The journey from symptom onset to diagnosis can be fraught with uncertainty and stress, exacerbated by the rarity of these conditions and the limited availability of tailored healthcare resources. Patients often face prolonged diagnostic pathways, repeated misdiagnoses, and inconsistent access to specialized care. These difficulties underscore the importance of collaborative research, patient advocacy, and policy development to address the unmet needs in the rare cancer community.

Despite advancements in oncology, the diagnosis and management of rare cancers, including MC-SI, remain hindered by several factors. These include a lack of characteristic symptoms, limited access to advanced diagnostic tools, and the inherent rarity that limits physician familiarity and clinical research. MC-SI often presents with vague clinical features such as abdominal pain, anemia, or weight loss—symptoms that mimic more common conditions, further delaying its identification. Imaging studies, histopathology, and immunohistochemistry are crucial for distinguishing this malignancy, but even these techniques require significant expertise to interpret findings accurately. Moreover, due to the limited prevalence of MC-SI, standardized diagnostic protocols are lacking, leaving clinicians reliant on case reports and small-scale studies to guide their decision making. The development of comprehensive diagnostic algorithms could greatly benefit this field, particularly if integrated with emerging technologies such as artificial intelligence and molecular diagnostics.

The COVID-19 pandemic, which began in late 2019, further exacerbated existing challenges in cancer diagnostics and treatment. Global healthcare systems were overwhelmed, and the focus on addressing the pandemic led to disruptions in routine medical services, including cancer diagnostics [4]. For many patients, delays in accessing healthcare facilities or undergoing diagnostic tests were compounded by fears of infection, resulting in a significant decline in cancer screenings and early-stage cancer detections [5]. Studies conducted in Europe, the United States, and Asia have demonstrated that these delays were associated with later-stage cancer diagnoses and worse prognoses for patients [6,7].

In the case of rare cancers such as medullary carcinoma of the small intestine, the pandemic’s impact was even more pronounced. The nonspecific symptoms of this malignancy, such as abdominal pain, anemia, or weight loss, often mimic more common gastrointestinal conditions, further complicating its diagnosis [3,8]. Additionally, the pandemic’s focus on respiratory symptoms led to an overdiagnosis of COVID-19 in patients presenting with symptoms such as coughing or weakness, delaying the identification of underlying conditions like cancer.

This study aims to highlight the diagnostic challenges of medullary carcinoma of the small intestine during the COVID-19 pandemic through a detailed case analysis. By examining the patient’s diagnostic journey, including the role of advanced imaging and immunohistochemical techniques, we provide insights into the critical factors necessary for accurate and timely diagnosis of this rare malignancy. Furthermore, we discuss the broader implications of pandemic-related diagnostic delays and emphasize the need for maintaining robust cancer diagnostic pathways during global health crises. Additionally, we explore potential strategies for improving the diagnostics and therapeutics for rare cancers, emphasizing the importance of innovation, multidisciplinary collaboration, and resilience in healthcare delivery systems.

## 2. Materials and Methods

### 2.1. Case Description

This study focuses on a 70-year-old male patient who initially presented to a family physician with symptoms of weakness, persistent cough, and respiratory discomfort. Routine diagnostic tests confirmed a positive SARS-CoV-2 result, and the patient was outpatient-treated for COVID-19. Despite completing the treatment, his symptoms did not resolve. Over the following months, the patient underwent a series of diagnostic procedures, including imaging studies, laboratory tests, and surgical intervention, culminating in the diagnosis of medullary carcinoma of the small intestine.

### 2.2. Data Analysis

The diagnostic process and its delays, which were exacerbated by the COVID-19 pandemic, were analyzed qualitatively to assess their impact on the patient’s outcomes. The histopathological findings were documented and compared with the existing literature on rare small intestinal malignancies to provide a broader context for the clinical significance of this case.

## 3. Results

### 3.1. Diagnostic Process

After recovering from COVID-19, the patient underwent a follow-up chest X-ray due to persistent respiratory symptoms. The imaging showed pleural fluid accumulation and lung parenchymal thickening, raising concerns about potential inflammatory or neoplastic processes. Laboratory tests performed during this period revealed significant anemia, prompting further investigation into potential underlying causes.

The patient’s laboratory results showed significant deviations from the norm, shedding light on the patient’s clinical condition and the advanced nature of the disease process. Below is a detailed description and interpretation of these results, integrated with the clinical findings given in Table 1.

A hemoglobin (Hb) result of 6.0 g/dL (reference range: 14.0–18.0 g/dL) indicated severe anemia, probably related to chronic bleeding, potentially secondary to malignancy. The anemia probably contributed to the patient’s general weakness and fatigue. A reduced red blood cell count (1.89 × 10^3^/µL (reference range: 4.5–5.9 × 10^3^/µL)) confirmed anemia, potentially resulting from iron deficiency associated with chronic inflammation or blood loss. A markedly elevated white blood cell count (32.17 × 10^3^/µL (Reference range: 4.3–10 × 10^3^/µL)) suggested an intense inflammatory reaction or the presence of a disseminated malignancy with associated inflammatory changes. Normal platelet levels (268 × 10^3^/µL (Reference range: 150–400 × 10^3^/µL)) indicated that coagulation pathways were not directly impaired at the time of the study. Elevated levels of both tumor markers—CEA (17.8 ng/mL) and CA 19-9 (60.80 U/mL)—indicated advanced malignancy. Hyponatremia (Sodium (Na): 128.4 mmol/L (Reference range: 132.0–146.0 mmol/L)) could be a sign of inflammation, dehydration, or renal dysfunction. Extremely high levels of urea (211.0 mg/dL) and creatinine (6.38 mg/dL) indicated severe renal dysfunction, which could be secondary to malignancy, dehydration, or systemic complications. Elevated CRP (35.5 mg/L (Reference range: <5.0 mg/L)) indicated the presence of an active inflammatory or neoplastic process. Elevated troponin (83 pg/mL (Reference range: <14 pg/mL)) suggested myocardial damage, potentially due to metabolic stress, severe anemia, or systemic effects of malignancy. These laboratory findings confirmed the critical condition of the patient.

During hospitalization, bronchoscopy was conducted to explore the respiratory symptoms further. Samples of bronchial lavage fluid and pleural fluid were collected for cytological analysis. However, no cancer cells were detected. Despite these findings, the patient’s symptoms persisted, necessitating additional imaging studies. However, following the implementation of basic medical interventions—adequate supplementation, hydration, and fluid removal—the patient’s clinical condition improved. It was concluded that renal dysfunction and signs of myocardial damage were likely secondary to the systemic disease and the overall deterioration of the patient’s condition. Hyponatremia and metabolic disturbances further complicated the clinical picture, reflecting a poor prognosis. After an improvement in the general clinical condition, supported by laboratory results such as a decrease in CRP (to 27.8 mg/L), WBC (12.36 × 10^3^/μL), and HGB (10.8 g/dL), the patient was discharged from the hospital with a recommendation for consultation and laboratory tests within the framework of primary medical care.

A computed tomography (CT) scan of the abdomen and pelvis was performed after the patient’s readmission to the hospital following a traffic accident. The CT findings included multiple focal lesions in both lobes of the liver, raising suspicion of metastases, as well as a hypodense lesion in the spleen. Enlarged lymph nodes were observed in the epigastric region, while segmental thickening of the small intestinal wall, with blurred boundaries and increased density in the surrounding adipose tissue, suggested potential neoplastic or inflammatory changes.

Based on the imaging findings, a decision was made to perform segmental resection of the affected small intestine. During the surgery, a 16 cm section of the intestine and 3 cm of associated mesentery were excised and sent for histopathological examination.

The described laboratory and clinical test results highlighted the need for a multidisciplinary approach to the patient’s treatment. This included advanced oncological therapy and supportive measures to enhance the quality of life. Early detection and intervention were critical in such cases to optimize treatment outcomes, particularly in the context of rare malignancies like medullary carcinoma of the small intestine. Unfortunately, the earlier diagnosis of SARS-CoV-2 infection in the outpatient setting delayed further diagnostics. Respiratory symptoms, the temporary improvement in the patient’s clinical condition, and the prioritization of efforts to combat the pandemic contributed to this delay in the diagnostic process.

### 3.2. Histopathological Examination

The gross examination of the resected intestinal segment revealed a tumor measuring 4.0 × 6.0 × 0.6 cm, with clear surgical margins free of invasive tumor cells. The cross-section revealed solid cancerous infiltration extending into the peri-intestinal fat tissue. No bacterial or viral infections were found in the tissues examined.

Microscopic analysis was performed on tissue samples prepared with hematoxylin and eosin (H&E) staining. The histological evaluation showed a solid growth pattern, consisting of uniform polygonal cells with amphophilic cytoplasm, prominent nucleoli, and occasional mitotic figures. These findings were consistent with medullary carcinoma.

To confirm the diagnosis, immunohistochemical staining was conducted using a panel of antibodies. Positive staining was observed for calretinin, pancytokeratin, and cytokeratin 7, while negative results were noted for cytokeratin 20, CDX2, CD56, S100, and synaptophysin. These results ruled out other malignancies, such as adenocarcinoma and neuroendocrine carcinoma, confirming the diagnosis of medullary carcinoma of the small intestine.

**Figure 1:**
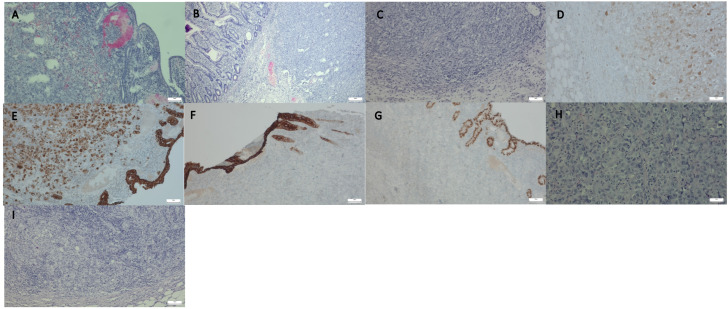
Histopathological images of medullary carcinoma of the small intestine and its metastases. (**A**,**B**): Infiltration of neoplastic cells in resected small intestinal tissue, showing uniform, polygonal cells with prominent nucleoli (H&E, 40×, 100×); (**C**): Lymphocytic infiltration accompanying the tumor (H&E, 100×); (**D**,**E**) Positive immunohistochemical reaction for calretinin and cytokeratin 7 in tumor tissue (small intestine) (100×, brown staining); (**F**,**G**): Negative reaction for cytokeratin 20 and CDX2 in tumor tissue (small intestine) (100×); (**H**): Metastatic medullary carcinoma in liver tissue (200×); (**I**): Metastatic medullary carcinoma in lymph node (100×).

Histopathological examination of the resected small intestine samples revealed solid cancer infiltration composed of uniform polygonal cells with amphophilic cytoplasm, prominent nucleoli, and occasional mitotic figures (Figure 1A,B). The cancer also infiltrated the peri-intestinal fat tissue, and lymphocytic infiltration was observed accompanying the tumor (Figure 1C).

It is worth noting that the patient’s initial diagnosis included a COVID-19 infection, which may have influenced the clinical presentation and test results. The COVID-19 infection could have contributed to the deterioration of the patient’s general condition, including worsening symptoms such as coughing and weakness, which might also have been the result of the developing neoplastic process. Additionally, the radiological changes in the lungs, such as parenchymal thickening and pleural effusion, were initially interpreted as potentially inflammatory, but it cannot be ruled out that they were partially related to the neoplastic process or a secondary effect of the infection.

Immunohistochemical staining showed positive expression for calretinin, pancytokeratin, and cytokeratin 7, consistent with the diagnosis of medullary carcinoma (Figure 1D–F). Negative results were obtained for cytokeratin 20, CDX2, CD56, S100, and neuroendocrine markers such as synaptophysin, which excluded other tumor types, including neuroendocrine carcinoma and adenocarcinoma (Figure 1G,H).

Based on these findings, a diagnosis of medullary carcinoma of the small intestine with metastases to the lymph nodes and liver, a fragment of which was also examined, was confirmed (Figure 1I). Based on histopathological examination and TNM classification, the tumor was staged as pT3N1M1, indicating: pT3—Tumor invades through the muscularis propria into the subserosa or non-peritonealized perimuscular tissue; N1—Regional lymph node metastases were identified in 1–3 lymph nodes; M1—Distant metastases were confirmed in the liver. Additional findings included the absence of lymphovascular invasion (LVI(−)), blood vessel invasion (BVI(−)), and perineural invasion (NI(−)). The surgical margins were negative (R0), confirming complete tumor resection with no residual microscopic disease at the primary site.

This staging suggests a locally advanced tumor with regional lymph node involvement and confirmed distant metastases to the liver. The absence of vascular and perineural invasion, combined with clear surgical margins, may indicate a more favorable prognosis at the primary site. However, the presence of distant metastases and lymph node involvement necessitates further oncological evaluation and systemic treatment to address the advanced stage of the disease.

## 4. Discussion

Medullary carcinoma of the small intestine is an exceedingly rare malignancy, and its diagnosis poses significant challenges due to nonspecific clinical symptoms, such as abdominal discomfort, anemia, or weight loss. These symptoms often overlap with those of more common gastrointestinal conditions, leading to delays in diagnosis. In this study, we highlight a case of medullary carcinoma misdiagnosed initially as COVID-19 during the pandemic, which underscores the critical need for greater awareness and advanced diagnostic techniques for rare cancers.

Our findings align with existing research emphasizing the rarity and diagnostic complexity of medullary carcinoma of the small intestine. Neoplasms of the small intestine are rare and account for less than 5% of all gastrointestinal cancer cases [1,2]. Cancers of the small intestine are primarily of two etiologies: small bowel adenocarcinoma (SBA), which accounts for 40% of cases, and neuroendocrine tumors, which account for another 40%. Gastrointestinal stromal tumors, sarcomas, and lymphomas make up the remaining 20–25% [8,9,10,11]. The medullary carcinoma of the small bowel is exceedingly rare, with only a few cases described in the literature [3]. Previous studies have shown that this tumor is often misdiagnosed as poorly differentiated adenocarcinoma or neuroendocrine carcinoma due to its solid growth pattern and lack of glandular features [1,3]. Immunohistochemistry remains the cornerstone for distinguishing medullary carcinoma, with calretinin being a key marker. This case corroborates earlier findings that cytokeratin 7 and pancytokeratin are often positive, while markers such as CDX2 and synaptophysin are negative, helping exclude adenocarcinoma and neuroendocrine tumors, respectively [3,8].

The pandemic significantly disrupted cancer diagnostics globally. Delays in routine cancer screenings, decreased access to healthcare facilities, and the overwhelming focus on respiratory symptoms led to missed or delayed cancer diagnoses, as documented in studies across Europe and North America [4,5,6]. In the present case, the patient’s initial COVID-19 diagnosis delayed the identification of the underlying malignancy. Such scenarios were not uncommon during the pandemic, where respiratory symptoms overshadowed other potential diagnoses. This highlights the need for heightened clinical vigilance during health crises, ensuring that non-COVID-related conditions are not deprioritized.

Studies have shown that diagnostic delays during the pandemic were associated with advanced-stage cancer diagnoses and worse prognoses [11,12,13,14,15,16,17,18,19]. Although medullary carcinoma is inherently aggressive, early detection could improve patient outcomes by allowing for timely surgical resection and treatment. The diagnostic delay in this case—spanning several months—likely contributed to the presence of liver and lymph node metastases at the time of diagnosis.

This case underscores the broader implications of the pandemic on healthcare systems and highlights the need for maintaining robust diagnostic pathways, even during emergencies. Telemedicine emerged as a critical tool during the pandemic, but its limitations in facilitating cancer diagnoses, particularly for rare malignancies, were evident [4]. Developing integrated systems that prioritize advanced imaging and prompt follow-up for unresolved symptoms, regardless of pandemic conditions, could mitigate such delays in the future.

The economic burden of rare cancers is another critical factor that cannot be overlooked. The high cost of diagnostic tests, coupled with the expense of experimental or off-label treatments, can place significant financial strain on patients and healthcare systems. This highlights the need for policy-level interventions to subsidize rare cancer research and ensure equitable access to advanced diagnostic and therapeutic options. Encouraging collaboration between academic institutions, industry stakeholders, and patient advocacy groups could accelerate progress in this field and alleviate some of the economic barriers faced by patients.

In addition to healthcare system limitations, the scientific understanding of MC-SI remains fragmented. Few studies have comprehensively examined its molecular and genetic characteristics, leaving gaps in knowledge regarding its pathogenesis, prognostic markers, and potential therapeutic targets. Molecular profiling of MC-SI tumors could uncover actionable mutations or pathways, paving the way for personalized treatment approaches. Furthermore, integrating molecular diagnostics with immunohistochemical techniques may enhance diagnostic accuracy and enable earlier detection, which is crucial for improving outcomes in rare cancers.

Immunohistochemistry played a decisive role in confirming the diagnosis of medullary carcinoma in this case. The consistent use of immunohistochemical markers, such as calretinin and cytokeratin 7, was crucial in distinguishing this rare tumor from other malignancies. Moving forward, greater emphasis on molecular diagnostics, such as next-generation sequencing (NGS) or circulating tumor DNA (ctDNA) testing, could further enhance the detection of rare cancers, particularly in cases where access to histopathological resources is limited.

This study has several limitations. First, as a single case report, its findings may not be generalizable to broader populations. Additionally, the lack of molecular profiling of the tumor limits our understanding of its genetic characteristics and potential therapeutic targets. Future research should focus on:

▪ Conducting larger-scale epidemiological studies to better understand the incidence and clinical presentation of medullary carcinoma.
▪ Investigating the genetic and molecular underpinnings of this rare cancer to identify potential biomarkers for early detection.
▪ Exploring the integration of telemedicine with advanced diagnostic tools, such as remote imaging and AI-based diagnostic systems, to improve cancer care during global health crises.

## 5. Conclusions

Medullary carcinoma of the small intestine is an exceedingly rare subtype of gastrointestinal cancer, often presenting significant diagnostic challenges due to its nonspecific symptoms and complex histopathological features. This case report highlights the diagnostic difficulties encountered in a 70-year-old patient with medullary carcinoma, who was initially misdiagnosed with COVID-19. The diagnostic delays were further compounded by the ongoing pandemic, as respiratory symptoms overshadowed other potential causes and diverted clinical attention. Despite undergoing bronchoscopy and a series of diagnostic tests, the cancer was only confirmed after imaging revealed suspicious lesions in the liver, spleen, and small intestine.

One of the primary challenges in the microscopic diagnosis of this tumor lies in its solid growth pattern, which can closely mimic poorly differentiated adenocarcinoma, a more common malignancy of the small intestine. Furthermore, certain tumor forms may exhibit infiltrative nests of tumor cells or a trabecular growth pattern, along with prominent nucleoli and a characteristic “salt and pepper” chromatin appearance. These features may lead to a misdiagnosis of neuroendocrine tumors. Consequently, additional immunohistochemical tests, such as calretinin staining, play a critical role in distinguishing medullary carcinoma from other malignancies and preventing underdiagnosis.

Following the final diagnosis, the patient was referred to an oncology clinic in good general condition to initiate treatment for the primary malignancy.

This report underscores the importance of advanced diagnostic techniques, particularly in cases involving rare cancers. It also highlights the necessity of maintaining heightened clinical awareness during global health crises, which may mask or delay the detection of both rare and common malignancies. The COVID-19 pandemic significantly impacted timely cancer diagnoses by prioritizing infectious disease management, thus delaying critical evaluations and, in turn, affecting patient outcomes. Early recognition, timely intervention, and a multidisciplinary approach remain crucial to optimizing treatment and improving prognoses in patients with rare tumors like medullary carcinoma of the small intestine.

## Figures and Tables

**Table 1:** Patient lab results.

Parameter	Results	Reference Range
Hemoglobin (Hb)	6.0 g/dL	14.0–18.0 g/dL
Erytrocytes (RBC)	1.89 × 10^3^/µL	4.5–5.9 × 10^3^/µL
Leukocytes (WB)	32.17 × 10^3^/µL	4.3–10 × 10^3^/µL
Platelets (PLT)	268 × 10^3^/µL	150–400 × 10^3^/µL
International Normalized Ratio (INR)	1.53	0.8–1.2 (non-anticoagulated)
Prothrombin Time (PT)	19.1 s	-
Prothrombin Activity (%)	56%	70–120%
Activated Partial Thromboplastin Time (APTT)	27.3 s	28.6–38.2 s
APTT–PTT Ratio	0.82 ratio	-
Amylase	78.5 U/L	28-100 U/L
Bilirubin (Total)	0.32 mg/dL	<1.2 mg/dL
Thyroid-Stimulating Hormone (TSH)	2.670 U/mL	0.27–4.2 U/mL
CEA	17.8 ng/mL	Smoke 0.0–4.4 ng/mL non-smoke 0.0–3.4 ng/mL
PSA (Total)	0.779 ng/mL	0.0–4.0 ng/mL
CA 19-9	60.80 U/mL	<39.0 U/mL
Folic Acid	2.61 ng/mL	3.8.0–16.0 ng/mL
Vitamin B12	760 pg/mL	191.0–663.0 pg/mL
Ferritin	47.1 ng/mL	30.0–400.0 ng/mL
HBsAg (Hepatitis B surface antigen)	negative	
Anti-HBc Total	negative	
Anti-HBs	<2 IU/L	Non-reactive [<10.0]
Anti-HCV	negative	
Glucose	121 mg/dL	82.0–115.0 mg/mL
Sodium (Na)	128.4 mmol/L	132.0–146.0 mmol/L
Potassium (K)	4.67 mmol/L	3.5–5.1 mmol/L
Chloride (Cl)	80.0 mmol/L	98.0–107.0 mmol/L
Urea	211.0 mg/dL	16.6–48.5 mg/dL
Creatine	6.38 mg/dL	0.67–1.17 mg/dL
eGFR	4 mL/min/1.72 m^2^	
CRP (Quantitative)	35.5 mg/L	<5.0 mg/L
ALT	12.6 U/L	<50.0 U/L
AST	40.4 U/L	<50.0 U/L
Troponin T-hs (STAT)	83 pg/mL	<14 pg/mL
pH	7.472	7.32–7.38
pCO_2_ (Partial CO_2_)	32.7 mmHg	35.0–48.0 mmHg
HCO_3_^−^ (Bicarbonate)	23.4 mmol/L	23–27 mmol/L
Base Excess (BE)	−0.30 mmol/L	−2.5–2.5 mmol/L

## Data Availability

The data presented in this study are available on request from the corresponding author due to the nature of the research, which describes a clinical case. Access to the data requires compliance with GDPR (General Data Protection Regulation) guidelines to ensure the anonymization and protection of sensitive personal information. Researchers interested in accessing the data are encouraged to contact the corresponding author to discuss the conditions under which access can be granted.
